# Recent Applications of Deep Learning Methods on Evolution- and Contact-Based Protein Structure Prediction

**DOI:** 10.3390/ijms22116032

**Published:** 2021-06-02

**Authors:** Donghyuk Suh, Jai Woo Lee, Sun Choi, Yoonji Lee

**Affiliations:** 1 Global AI Drug Discovery Center, School of Pharmaceutical Sciences, College of Pharmacy and Graduate, Ewha Womans University, Seoul 03760, Korea; dsuh@ewha.ac.kr (D.S.); jwl123@ewha.ac.kr (J.W.L.); sunchoi@ewha.ac.kr (S.C.); 2 College of Pharmacy, Chung-Ang University, Seoul 06974, Korea

**Keywords:** structural bioinformatics, deep learning, protein sequence homology, 3D structure of proteins, drug discovery

## Abstract

The new advances in deep learning methods have influenced many aspects of scientific research, including the study of the protein system. The prediction of proteins’ 3D structural components is now heavily dependent on machine learning techniques that interpret how protein sequences and their homology govern the inter-residue contacts and structural organization. Especially, methods employing deep neural networks have had a significant impact on recent CASP13 and CASP14 competition. Here, we explore the recent applications of deep learning methods in the protein structure prediction area. We also look at the potential opportunities for deep learning methods to identify unknown protein structures and functions to be discovered and help guide drug–target interactions. Although significant problems still need to be addressed, we expect these techniques in the near future to play crucial roles in protein structural bioinformatics as well as in drug discovery.

## 1. Introduction

Proteins, large and complex polymers with linear amino acid chains, play crucial roles in cells responsible for constructing and regulating our body. By revealing the structure and contacts of biomacromolecules, we gain a better understanding of their function, thus facilitating the rational drug discovery process. The recent advances in experimental structural biology techniques such as X-ray crystallography, nuclear magnetic resonance (NMR), and cryogenic electron microscopy (cryo-EM) have fueled accurate structure determination [1,2,3,4]. However, owing to the high cost and time-consuming aspects of experimental determination, there is still a large “structure knowledge gap” between the vast amount of protein sequences and a relatively small number of known structures. Therefore, knowledge-based theoretical techniques to elucidate protein structure are in need. After Anfinsen’s dogma stating that the native structure of at least a small globular protein is determined by the sequence only, various attempts to identify protein structure from its sequence have been made, starting with predicting folding states of protein by Pauling and Corey in 1951 [5,6,7]. The significant breakthrough in next-generation sequencing (NGS) technology has led to burgeoning sequence information, and a fundamental problem in structural bioinformatics is predicting 3D structures using these tremendous sequence data [8].

Protein structure prediction has become more powerful and accurate with method developments from traditional statistical methods to machine learning (ML) and deep learning (DL) methods [9,10,11]. Artificial neural network, especially deep neural network, is a good fit for protein structure prediction with its ability to express a wide variety of functions and its efficiency relying heavily on the amount of quality data. The introduced concepts of homology and evolutionary information empowered the process, and the advent of robust equipment such as graphical processing unit (GPU) expedited it [12,13,14]. Initially, some pieces of protein structures such as helical status or torsional angles are targeted for prediction, and then the whole structure is deduced utilizing the predicted features known as protein structure annotations (PSAs) [15,16]. In order to catch up with recent progress and to know the state-of-the-art method, one can check with Critical Assessment of Structure Prediction (CASP), a worldwide community experiment held every two years to assess the effectiveness of prediction methods [17,18,19]. The usage of the artificial neural network not only saves time and cost, but also strengthens the functional analysis of large-scale proteomics studies. ML and DL technologies based on various computational methods enable the detection of protein–protein interaction (PPI) in heterogeneous types of proteomics data [20]. Multi-faceted analysis of protein structures can be linked to the prediction of drug–target interaction (DTI) [21]. As the application of deep learning methods to drug discovery areas is at a nascent stage, various machine and deep learning methods need to be considered and tested for better accuracy in analyzing PPIs.

In this review, we will provide an overview of DL-associated protein structure prediction, related concepts, frequently used DL architectures, and developed methods predicting various PSAs delineating different levels of details of protein structure. The further applications of DTI are of interest and discussed. Finally, current limitations, as well as the advantages of DL-based protein structure prediction upon drug discovery field, will be highlighted.

## 2. Protein Sequence Homology, 3D Structure, and Deep Learning

### 2.1. Protein Sequence Homology

The central dogma of molecular biology states that DNA sequences are transcribed into messenger-RNA (mRNA), and then these mRNA sequences are translated into protein sequences. Searching similar sequences can be used to reveal “homologous” genes or proteins by detecting statistically significant similarity, which indicates common ancestry. This protein sequence, in structural biology, is assumed to determine the three-dimensional structure and function of a protein. It is based on the fundamental observation that similar sequences from the same evolutionary family will typically adopt similar protein structures. Moreover, the structures of proteins are highly conservative in evolution compared with their sequences, and the number of unique structural folds is generally thought to be limited in nature. Thus, tremendous effort has been put into quarrying the relationship between structure and sequence of proteins. As the number of protein sequences is exponentially increasing, while the experimentally verified structures are growing slowly, we expect homology-based contact map prediction and modeling to become far more popular.

### 2.2. 3D Structural Space of Proteins

A protein structure can be defined as one of four levels: primary, secondary, tertiary, or quaternary structures. Primary structure is a linear sequence of amino acids. There are 20 standard amino acids available to form a protein, and each amino acid is connected to the next one via peptide bonds. Primary structure is often introduced as a string of letters, i.e., ‘AESVL…’, as each standard amino acid has a corresponding single-letter code (and three-letter code). This already gives much useful information with respect to protein structure in three-dimensional space owing to the distinctive characteristics of each amino acid. For example, the different hydrophobicity of each amino acid limits the conformation of the protein, and some unique covalent bonds can be formed only between certain amino acids such as cysteine. Many ab-initio protein structure predictions start with this sequence of amino acids, a primary structure.

Secondary structure defines the form of local segments of proteins. It is normally determined by the hydrogen bond patterns of polypeptide backbone or backbone dihedral angles (φ,ψ). The two common secondary structures are α-helices and β-strands. An α-helix is a segment of amino acids where the main chain forms a helix, pointing side-chains outward. Two hydrogen bonds per residue stabilize this helix formation. A β-strand is rather connected laterally where side chains are pointing out perpendicularly to the plane with each successive residue facing the opposite side. This form normally requires a partner β-strand unit for its stability. When used in protein structure prediction, secondary structure normally falls into a three-state or a fine-grained eight-state categorization. The three-state categorization consists of two regular types of α-helix (H) and β-strand (E), and one irregular type of coil region (C). The widely-used eight-state categorization based on the Dictionary of Secondary Structure for Proteins (DSSP) program by Sanders further dissect helices into three types as 3_10_ helix (G), α-helix (H), and π-helix (I); strands into two types as β-strand (E) and β-bridge (B); and coils into three types as β-turn (T), high curvature loop (S), and any other previously undefined type (C) [22].

Tertiary and quaternary structures elucidate a three-dimensional arrangement of the single and multiple proteins, respectively. They can be represented using the Cartesian coordinates of each atom in three-dimensional space. Owing to the aqueous nature of proteins, the main driving force deciding tertiary and quaternary structure is the hydrophobic interaction among amino acids and water molecules. Thus, proteins tend to possess a hydrophobic core where side chains are buried, avoiding polar water molecules. Such three-dimensional information is deducible when one has the primary structure, secondary structure, and inter-residue CM in hand.

In contrast to sequences, which are virtually infinite in number, proteins can take on a finite number of different shapes to carry out their functions in the cell. One can observe stronger structural conservation than sequence conservation; for example, a strong interdependence for polar residues exists at protein core with poor solvent accessibility, but no significant correlation is detected when looking at sequences only [23]. This makes it feasible to predict protein structure, a more conserved domain, from abundant sequence data [24]. Hence, various attempts to unravel the relationship between the structure and sequence have been made, including deep learning methodologies and pre-eminent approximations for underlying mapping functions.

### 2.3. Overview of Deep Learning Methods

Deep learning is a branch of machine learning, utilizing an artificial neural network with many layers embedded, which resembles a human nervous system. Working as universal function approximators, deep neural networks are used to solve various problems: classification, clustering, pattern recognition, predictive analysis, regression, and so on [25]. With the rapid and tremendous growth of biomedical data sources, deep learning can be applied to multi-omics data analysis, disease categorization, and healthcare social network analysis. It indicates that high-quality data that are used to train and build deep learning models should be appropriately labelled for biomedical data analysis. When high-quality datasets are available for deep learning models, reproducible deep learning models can be built to analyze newly collected biomedical data of similar structures.

**Figure 1:**
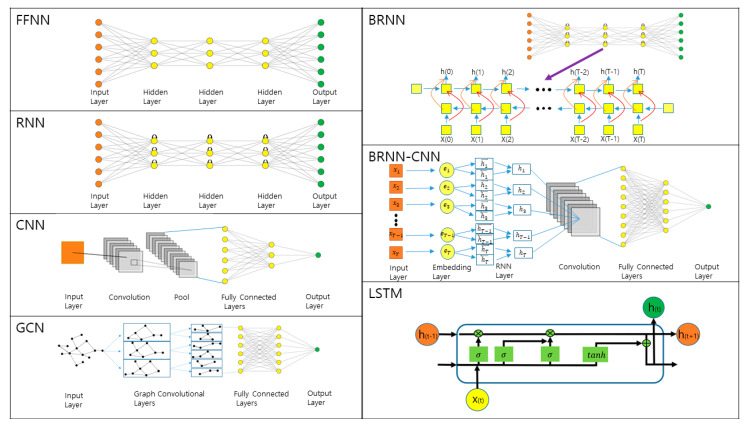
Overview of deep learning (DL) architectures frequently used for protein structure prediction.

Artificial neural networks consist of nodes in input, output, and hidden layers where each node is connected to nodes in adjacent layers. These connections have distinct weights, and the inputs are processed (i.e., multiplication and summation) at each node. Then, it undergoes the transformation based on the activation function such as sigmoid or rectifier, and the output functions as the input for the next layer. Learning is the process of finding optimal weights that make the neural network behave as desired. Two types of learning are present; supervised learning handles labeled datasets for classifying or predicting purposes, while unsupervised learning handles unlabeled datasets for analyzing or clustering the given dataset. The amount of required training data to build effective deep learning models is dependent on the complexity and the number of features in the training data. To update and optimize the weights, back-propagation is used to calculate the gradient of the loss function that computes the error for each training iteration [26]. When too many layers are used, however, the gradients either vanish or explode, making the training process inefficient [27]. Certain tricks such as modifications upon activation functions (i.e., rectified linear unit (ReLU)) and utilizations of skip connections (i.e., residual neural network) exist to overcome this issue [28,29,30]. With these steps as a fundamental basis, there are miscellaneous architectures for artificial neural networks. With the expanding data availability for protein sequences and structures of closely related homologs, deep learning methods have been presented for protein structure prediction, and a few frequently used architectures will be discussed in this section (Figure 1).

The most straightforward and earliest stage example for the deep neural network is the feedforward neural network (FFNN), sometimes called multilayer perceptron (MLP). A perceptron, a single-layer neural network, can only process first-order information to obtain results comparable to those obtained by multiple linear regression. When multiple layers are used, the neural networks can extract higher-order features. In FFNN, information flows in one direction from the input layer to hidden layers, if any, until it reaches the output layer. The network has connections between each node and every other node in the next layer.

Recurrent neural network (RNN) contains loops where the output of the layer becomes an input. This looping generates state neurons that enable the network to possess memory about the previous state. Obtaining a future memory is favorable for prediction and is feasible with RNN by introducing a delay, but the prediction rates drop if the delay is too large. To overcome this issue, a bidirectional recurrent neural network (BRNN) has been developed, splitting the state neurons into positive and negative time directions [31,32]. 2D-BRNN, a two-dimensional application of BRNN, has been widely used to correctly predict the residue contact map (CM), normally using four-state vectors handling four cardinal corners of the map [33]. Long-short term memory (LSTM) is a variant of unit cell used in RNN, designed to resolve vanishing gradient problems by introducing gate functions into the unit cell [34]. This error gating allows LSTM to learn long-term dependencies between data points. With their ability to permit sequence as inputs and outputs, RNNs are known for excellent performance upon any sequence-based problems, suitable for protein structure prediction with protein sequence as input.

Convolutional neural network (CNN) often encompasses three types of layers: convolutional, pooling, and fully connected layers [35,36]. CNN generally takes input such as a 2D image, and the convolutional layers apply various kernels to convolve it where each kernel acts like a perceptron, generating feature maps. Then, a pooling layer follows to perform dimension reduction upon the network parameters and feature maps. The results are forwarded into the fully connected layers, mapping 2D feature maps into a 1D vector for further feature representations. The main benefit of applying the convolution scheme is the massive parallelism, yielding a great amount of computational efficiency. Convolutional schemes are widely used for CM prediction, a 2D-PSA [37].

Graph deep learning models enjoy attention from numerous application domains thanks to their structural consistency to the native graph-structured data. Graph convolutional network (GCN), a generalization of the convolutional operator upon non-Euclidian structured data, contains several spectral or spatial convolutional layers [38]. Its unique featurization strategies at the input level with elaborated architectures suit complicated problems such as PPI or DTI.

To improve our fundamental understanding of biological phenomena, protein structures and their contacts shed light on their mechanism of action, possibly assisting with drug design. Based on the co-evolution analysis and deep learning methods, protein structure prediction methods have made significant progress in recent years by using multiple sequence alignments (MSAs) of the target protein and its homolog. A combination of the architectures mentioned above is widely used in this type of protein structure prediction methods. One famous example would be a combination of bidirectional RNN and CNN (BRNN–CNN) [39]. In this scheme, a convolutional kernel maps a window of BRNN memories into a local state. There exist variations such as bidirectional LSTM followed by CNN (BLSTM–CNN) [40]. Unlimited hybrid topologies are available, but one needs to design the architecture carefully, considering training difficulty, computational complexity, and memory requirement in order to obtain the best accuracy.

## 3. Prediction of 1D and 2D Protein Structural Annotations

Proteins and their functions are distinguished by their structures in numerous aspects, but the rate of discovering protein structures has been much slower than the rate of sequence identifications owing to the cost and complexity. Therefore, protein structure predictor has become one of the most efficient and high-throughput tools in Bioinformatics to handle flooding known sequence data with developing methodologies such as statistical, ML, and DL methods. The feature used in the predicting process is known as PSA; it contains simplified information to ease the computing process and is used as an intermediate step to estimate the full protein structure. One dimensional- (1D-) and two-dimensional- (2D-) PSAs have enjoyed a great amount of attention, where secondary structure, solvent accessibility, or intrinsic disorder is mainly described as 1D-PSA, and CM or the detailed version of CM (multi-class CM or distance map) is expressed with 2D-PSA. Several DL applications have been developed for 1D- and 2D-PSA predictions, becoming more accurate owing to expanding of the availability of sequence and structure data.

### 3.1. 1D Prediction

The most fruitful feature among 1D-PSAs is the secondary structure, the very first step for the full protein structure prediction from the sequence. Two main classifications are available: three-state categorization into α-helix, β-strand, and coil region, or eight fine-grained categorizations, which further segregate the previous three states (vide supra). The earlier stage methods have used sequence data solely as input sources, but later, evolutionary information and physicochemical properties were involved in enhancing the prediction accuracy [41]. The accuracy can be easily expressed by three-state percentage accuracy (Q_3_ score) or eight-state percentage accuracy (Q_8_ score), which is defined as the percentage of correctly predicted secondary structure residues.

One of the earliest servers available for secondary structure prediction would be JPred developed by Cuff et al. [42]. The server adopts six different secondary structure prediction algorithms: DSC using linear discrimination, PHD using jury decision neural networks, NNSSP using nearest neighbors, PREDATOR using hydrogen bonding propensities, ZPRED using conservation number weighted prediction, and MULTIPRED using consensus single sequence method combination [43]. Another secondary structure prediction server, PSIPRED, became available, where the method conjugates two FFNNs, training neural networks upon evolutionary conservation information derived from PSI-BLAST [44,45]. Another attempt called SSpro showed an enhanced algorithm application, using BRNN–CNN [46]. The method utilizes a mixture of estimators that leverages evolutionary information, indicated in multiple alignments, both at input and output levels of BRNN. Porter, Porter+, and PaleAle among the Distill series are also based on ensembles of BRNN–CNN, each used to predict different 1D-PSAs (Porter for secondary structure prediction, Porter+ for local motif prediction, and PaleAle for residue solvent accessibility prediction) [47]. In the following Distill methods, the sequence is processed by the first BRNN–CNN stage and then pulled into a set of averages, which are processed by the second BRNN–CNN stage. Porter achieved better performance using both PSI-BLAST and HHBlits for harnessing evolutionary information [48,49]. Likewise, Porter+ considers local structural motifs for predicting torsional angles [50]. PaleAle, dealing with relative solvent accessibility (RSA), is structured with double BRNN–CNN stacks in the most recent version of 5.0, surpassing benchmarks from other methods for RSA prediction [51]. NetSurfP-2.0, concatenating CNNs and BRNNs, was developed in 2019. This method predicts secondary structures, solvent accessibility, torsion angles, and intrinsic disorder, all at once [52].

Taking other 1D-PSAs into account along with secondary structure and considering physicochemical properties, as well as evolutionary information, helped to enhance the overall accuracy. DESTRUCT, proposed by Wood and Hirst, iteratively used cascade-correlation neural networks upon both secondary structure and torsional angles [53]. The iteration is composed of the first FFNN trained to predict the secondary structure and φ dihedral, and filtering FFNN intervening successively to transform the predictions into new values. Hirst group upgraded DESTRUCT into DISSPred that relied on support vector machine (SVM) and obtained better performance [54]. SPINE-X by Faraggi et al. in 2012, later replaced by SPOT-1D from the same group, enhanced the accuracy by incorporating physicochemical properties such as hydrophobicity, polarizability, and isoelectric point, among others. This method could also be used for residue solvent accessibility and torsion angle predictions [55,56]. SPIDER2 launched anticipated multiple 1D-PSAs—secondary structure, solvent accessible surface area (SASA), and torsion angles—all at once with three iterations of deep neural networks [57]. Its successor, SPIDER3, improved the performance overall, and now the method predicts four PSAs at once, including contact number with four iterations for the prediction [58]. ProteinUnet, published in 2020, yields similar accuracy for secondary structure prediction as SPIDER3-single, but uses half parameters with an 11-fold faster training time [59,60]. Most servers and methods discussed now have over 84% Q_3_ score in their latest versions with deeper neural networks and better algorithms. Considering the explosive advancement in reliability for Q_3_ score with DL methods, it might not take too long until the theoretical limit of 88–90% is attained.

One special kind of 1D-PSA targets disordered regions of proteins. Many proteins contain intrinsically disordered regions (IDRs) that are highly flexible. Having multiple structures available, IDRs are involved in assembling, signaling, and many genetic diseases [61]. Therefore, this PSA is of particular interest in addition to being a component of full protein structure prediction. IDRs have been predicted using statistical potentials, SVM, or artificial neural networks. IUPred employs a statistical pairwise potential expressed as a 20 × 20 matrix that expresses the general preferences of each amino acid pair in contact [62]. The pairwise energy profile is calculated, and disorder probability is estimated accordingly. DISOPRED3 method is formulated on SVM, a supervised machine learning model, to discriminate between ordered and disordered regions [63]. DISOPRED3 is trained on PSI-BLAST profile because it outperforms the models trained on single sequences, showing the improvements predicated on evolutionary information. SPOT-Disorder2 offers per-residue disorder prediction based on a deep neural network utilizing LSTM cells [64]. Higher accuracy was obtained by upgrading its architecture from a single LSTM topology used in the previous version, SPOT-Disorder, to an ensemble set of hybrid models consisting of residual CNNs with inception paths followed by LSTM layers [65].

### 3.2. 2D Prediction

With the information gained from 1D-PSAs in hand, one might need 2D-PSAs to fully construct the three-dimensional protein structure. Recent endeavors for 2D-PSAs are focused on CM and multi-class CM, both expressing the closeness between residue pairs in a protein. CM takes a binary 2D matrix structure of N × N, where N is the length of the protein sequence, assessing each residue pair as 1 (presence) or 0 (absence) for matrix elements based on the user-defined threshold Euclidean distance (a typical value is ~8 Å between Cα atoms). Multi-class CM is expressed in a 2D matrix, but the matrix elements are quantized in detail, categorized into more than two states. The importance of this CM for protein structure prediction is directly shown in estimations; an early study estimated that one could assemble a structure model within 5 Å RMSD from the native structure if N/4 long-range protein contacts are known, and another study estimated that one contact per twelve residues allows for robust and accurate protein fold modeling [66,67].

The CM itself definitely provides useful information on the given protein’s spatial organization, but one should note that CMs often contain transitive noise coming from “indirect” correlations between residues. Methods for direct correlation analysis are used to remove this noise such as mutual information (MI), direct coupling analysis (DCA), and protein sparse inverse covariance estimation (PSICOV) [68,69,70]. DCA infers direct co- “evolutionary couplings” among residue pairs in an MSA table to uncover native intra-domain and inter-domain residue–residue contacts in protein families [71,72].

Many groups have developed CM predictors utilizing multi-stage deep neural networks. The previously introduced Distill server also provides the CM predictor named XX-Stout [47]. The developers included contact density profile as an intermediate step using another Distill module named BrownAle [73]. Calculating this contact density profile, principal eigenvector significantly increased the performance overall. DNCON by Eickholt and Cheng took advantage of surging GPU developments for training largely boosted ensembles of residue–residue contact predictors [74]. MetaPSICOV is another CM predictor known for the first method utilizing co-evolution signals from 1D-PSAs extracted with three different algorithms [75]. Then, a two-layer neural network was used to deduce CM. Its successive versions, named MetaPSICOV2 and DeepMetaPSICOV, exist where deeper network architecture and ReLU units are employed. RaptorX-Contact from RaptorX series utilized co-evolution signals to improve the accuracy [76]. RaptorX-Contact predicts local structure properties, contact and distance matrix, inter-residue orientation, and tertiary structure of a protein using an ultra-deep convolutional residual neural network from primary sequence or a multiple sequence alignment. DNCON2 is implemented with six CNNs and applied co-evolution signal from 1D PSAs. This method predicts CM with various distance thresholds of 6, 7.5, 8, 8.5, and 10 Å, and then refines them to leave with only 8 Å CM with an improved prediction rate [77]. TripletRes starts with the collection of MSAs through whole-genome and metagenome sequence databases and then constructs three complimentary co-evolutionary feature matrices (covariance matrix, precision matrix, and pseudolikelihood maximization) to create contact-map models through deep residual convolutional neural network training [78]. DeepContact is also a CNN-based approach that discovers co-evolutionary motifs and leverages these patterns to enable accurate inference of contact probabilities [79]. The authors argue that the program is useful, particularly when few related sequences are available. DeepCov uses fully convolutional neural networks operating on amino-acid pair frequency or covariance data derived directly from sequence alignments, without using global statistical methods such as sparse inverse covariance or pseudolikelihood estimation [80]. In contrast to other software programs that require third-party programs, Pconsc4 is a hassle-free contact prediction tool that does not use any external programs [81].

Recently, in 2019, DeepCDPred was developed, which includes a multi-class CM predictor exploiting distance constraint terms [82]. The authors used four FFNN-based models to distinguish four classes of contact ranges: 0–8, 8–13, 13–18, and 18–23 Å. AlphaFold from the same year generates the most fine-grained multi-class CM, 64 equal bins distogram (distance histogram) along 2–22 Å, becoming state-of-the-art for the field [83]. An architecture of deep 2D dilated convolutional residual network with 220 residual blocks was employed for the distance map prediction in AlphaFold (note that it will be discussed in more detail in the next section). These 2D-PSA developments have benefitted from the growth of affiliated fields, including algorithmic development and advancement of technologies, which is immediately beneficial for precise 3D structure prediction. The prediction methods for protein structure annotations are summarized in Table 1.

## 4. Prediction of Protein 3D Structures

One of the main goals of structural bioinformatics is to unravel the relationship between the individual amino acids that make up a protein and the corresponding 3D structures, i.e., to distinguish the relationship between genotype and phenotype. A breakdown in this relationship may allow us to clarify the role of certain proteins, such as the binding to specific targets or catalyzing novel chemical reactions, providing insights into biological advances and drug discovery. Several experimental techniques for structure determination exist and have been continuously developing. Decades of theoretical work has attempted to predict protein structures from their amino acid sequences and, in some way, 1D- and 2D-PSA predictions are also the efforts to excel this 3D structure prediction. Significant progress has been achieved on this problem thanks to the rapidly growing number of available sequences and the application of global statistical methods. Deep learning has become the dominant technology to predict protein structures based on contact or evolutionary maps.

### 4.1. Critical Assessment of Protein Structure Prediction (CASP)

CASP (https://predictioncenter.org/index.cgi) is a biannual competition with global collaborative efforts designed to evaluate the state-of-the-art techniques in protein structure prediction. The algorithm for tertiary structure prediction can be subdivided into the following: homology modeling, which utilizes a known structure with a similar sequence as a template (template-based modeling, TBM); fold recognition, which is also called protein threading (templates required); and de novo structure prediction, which is template-free modeling. Recent advances in DL-related techniques have been increasing the accuracy of contact distance prediction and residue–residue co-evolutionary analysis and, finally, in the past several years, significant progress has been made in template-free protein structure prediction as well as template-based modeling [11,19].

### 4.2. 3D Structure Prediction Based on Contact Maps

Genomic sequences, the valuable resource of evolutionary information, can be efficiently mined to detect correlations or covariations between residues in proteins (so-called “evolutionary couplings”). Analyzing this covariation may help identify directly contacting residues in 3D conformations, functional residues in substrate binding, or residues involved in protein–protein interactions. As discussed in the earlier section, CM is a bi-dimensional matrix coding the absence/presence or the probability of contact between residue pairs in a given protein. The values near the main diagonal in a CM are trivial because these are the ones from adjacent amino acid pairs (note that adjacent residues should always have high contact probability). The most relevant information in a CM is located far from the main diagonal. Analyzing elements far from the main diagonal may give useful information about structural properties and spatial details of the protein backbone. Hence, these contacts or multi-class contact maps can provide information concerning the spatial organization of the protein and can be used to improve the quality of the predicted tertiary structure. In the case of a typical globular protein, nearly 90% of all residue pairs are expected to be non-contacting, so that only a small portion of inter-amino acid distances should be accurately employed as constraints to direct structure determination. Advanced deep learning techniques have shown promise in predicting accurate residue–residue contacts. In order to increase the accuracy of CM, one may need to consider several key factors such as deep learning techniques, reliable MSA, distance distribution prediction, and domain-based contact integration.

AlphaFold (Google DeepMind), the latest hot trend in this field, was first released at CASP13 (2018) and has evolved to AlphaFold2 at CASP14 (2020) [83,84]. DL with an attention algorithm trained the neural networks on ~170,000 known protein structures [85]. First, co-evolutionary analysis is used to match amino acid sequence covariations with physical contact on a protein’s 3D structure and is further examined using neural networks to examine the patterns of co-evolutionary interactions and convert them into CMs. Based upon evolutionarily related protein sequences and amino acid residue pairs, the model iteratively generates a structure by passing information back and forth between both representations. In AlphaFold1, the distance map is generated by the information from multiple sequence alignment and is used to produce a guide potential. A simple gradient descent technique is employed to directly fold the protein into a structure compatible with the predicted distances. Then, the Rosetta energy function is used to refine the final folded structure. The detailed process of AlphaFold2 is not published yet, but it seems that the guide potential process is replaced with a system entirely based on pattern recognition, and the energy refinement based on AMBER forcefield is applied as a final refinement step. The model achieved outstanding results with a median global distance test (GDT) score around or above 90 overall across all targets. The program was able to reliably predict the structures of membrane proteins that have been exceedingly difficult to solve until now.

**Figure 2:**
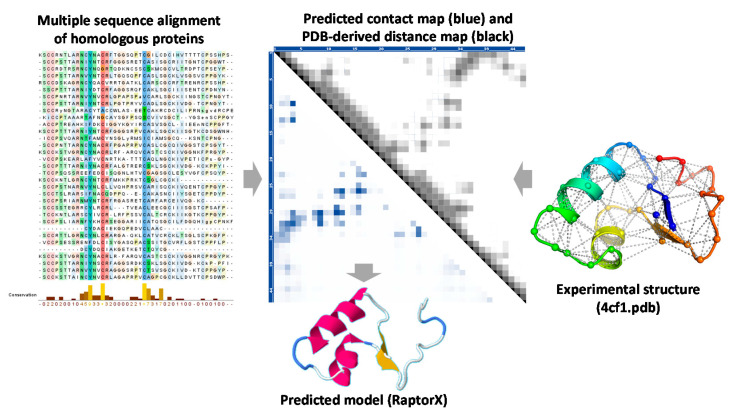
Contact map of the exemplary small protein Crambin (UniProt id: P01542). (**left**) Part of the multiple sequence alignment of the homologous proteins of Crambin. (**middle, lower half**) Contact map and the corresponding 3D model predicted by RaptorX. A contact is defined by Cβ-Cβ distance ≤ 8 Å. Darker color indicates a higher probability. (**middle, upper half**) Distance map based on the 3D experimental structure of the protein. The map was visualized using the VMD plugin. The Cα–Cα distance for each pair is plotted and colored black at 0.0 Å distance, to a linear grayscale between 0.0 and 10.0 Å, and white when equal to or greater than 10.0 Å. (**right**) X-ray crystal structure of Crambin (PDB id: 4fc1) overlaid with the contact (Cα-Cα, cutoff distance 8 Å) marked in gray dashed lines.

Although the AlphaFold series were more prominent than other competitors during the two CASP experiments, various other prediction programs were developed based on deep learning and showed significant progress. For example, RaptorX [76] is a server for protein structure and function prediction powered by DL. It predicts protein secondary and tertiary structures, solvent accessibility, disordered regions, functional annotation, and possible binding sites. It also provides inter-residue/inter-atom distance and orientation probability distribution that may be used by other folding algorithms to rebuild protein 3D models (Figure 2). In the program, the quality of MSA profiles is assessed by a profile-entropy scoring method considering the available non-redundant homologs. Then, conditional random fields are used to integrate a variety of biological signals in a nonlinear threading score function. Rosetta suite uses the algorithm for de novo structure prediction, also used in dealing with protein folding in divergent domains of homology models. Initial protein folding of short segments is selected from the protein structure database, whereas longer segments are constructed from three- and nine-residue fragments selected from the database and then assembled using the Rosetta algorithm [86]. SPOT-fold is a fragment-free ab initio protein structure prediction tool guided by predicted backbone structure and CM from SPOT-Contact, as well as by predicted dihedral angles from SPIDER3 [37,60,87]. DMPfold uses deep learning to predict inter-atomic distance bounds, the main chain hydrogen bond network, and torsion angles, which it uses to build models in an iterative fashion [88]. MULTICOM is a protein structure modeling server empowered by DL and contact distance prediction [89]. EVfold disentangles direct and indirect residue–residue correlations in large multiple sequence alignments and derives direct residue–residue evolutionary couplings [90]. They provide several modules such as EVcomplex to predict protein–protein interaction complex structure with evolutionary coupling analysis.

### 4.3. In Combination with Template-Based Modeling

One of the popular and successful approaches to protein structure prediction is homology modeling, which relies on two principles: (i) the amino acid sequence determines the protein’s typical fold or 3D structure, and (ii) the 3D structure is somehow preserved with regards to the primary sequences. Using known structures of homologous proteins that have a certain degree of sequence similarity, it is a quite convenient and effective way to build an initial model. However, the problems in the weak sequence–structure similarities, aligning sequences with structures, modeling of rigid body shifts, and accurate conformations of loops and side chains, as well as detecting errors in a model, are still recognized to date. The combination with DL-based approaches recently appears to outperform conventional methods, accomplishing a significant improvement in model accuracy. The CASP13 and 14 results demonstrate that the complex mapping between amino acid sequence and 3D protein structure can be effectively learned using a neural network and generalized to previously inaccessible cases.

The homology modeling is generally performed by the following steps: (i) identify and select the eligible templates, i.e., other homologous proteins with known 3D structures (related programs: BLAST, PSI-BLAST, HH-suite (HHsearch, HHblits, and HHPred), and JackHMMer, among others); (ii) multiple sequence alignment (related programs: CLUSTAL Omega, MUSCLE, and so on) [91,92]; (iii) 3D model building (related programs: SWISS-MODEL, MODELLER, I-TASSER, and so on) [93,94,95]; (iv) modeling of loops that are variable and not conserved region; (v) side-chain modeling based on rotamer library, a scoring function, and a scanning method (related programs: OPUS-Rota2, FASPR, SCWRL) [96,97,98]; (vi) model optimization increasing the quality of the final model (generally energy minimization, molecular dynamics, or Monte Carlo simulations); and (vii) model evaluation and validation.

DL-based methods can be employed to improve the accuracy in each step. For example, DLPAlign is an example of a DL approach combined with sequence alignment [99]. It is a novel and straightforward approach to improve the accuracy of the progressive MSA method by training a decision-making model based on convolutional neural networks. DESTINI (deep structural inference for proteins) is a novel computational approach that combines a deep-learning algorithm for protein residue/residue contact prediction with template-based structural modeling [100]. ThreaderAI first applies DL to predict residue–residue aligning probability matrix by integrating sequence profile, predicted sequential structural features, and predicted residue–residue contacts, and then utilizes dynamic programming to template-query alignment according to the probability matrix [101]. C-I-TASSER (contact-guided iterative threading assembly refinement) is an extended method from the original I-TASSER for high-accuracy protein structure and function predictions [102]. It generates inter-residue CMs using multiple deep neural-network predictors (such as NeBcon, ResPRE, and TripletRes) and identifies reliable structural templates from the PDB database by multiple threading approach (LOMETS) [78,103,104,105]. Then, the full-length atomic models are assembled by contact-map-guided replica-exchange Monte Carlo simulations. In the large-scale benchmark tests, C-I-TASSER generated significantly more accurate models than I-TASSER, particularly for sequences with no homologs in the public database.

DL methodologies are leading successes in co-operative fields, namely model quality assessment (QA), which is a succeeding step for protein structure prediction. For both template-based and template-free methods, QA is followed by structure predictions to measure the divergence from the natively folded protein structures. Since CASP7(2006), QA has been categorized for competition to develop methods for assessing the quality and correctness of protein structure models [106]. Earlier statistical methods including PROCHECK and WhatCheck focused on the stereochemistry of a protein structure such as backbone dihedral angles or non-bonded distances between residues [107,108]. The predicted models could also be evaluated using residue–residue interaction energies where peaks in the energy profile would mean erroneous prediction of the region. Later, DL-based methods for QA were developed and highlighted. AngularQA by Cao group utilized sequence properties like secondary structures in addition to angles upon QA problem, becoming the first attempt utilizing LSTM cells for QA problems [109]. GraphQA tackled QA problems with GCNs for desirable properties such as representation learning, geometric invariance, explicit modeling of 3D structure, and so on [110].

## 5. Prediction of Drug–Target Interactions (DTIs)

The identification of the physical interactions between new drug candidate molecules and their biomolecular targets is an essential part of designing new drugs. A computational approach with the ability to predict novel drug–target interactions (DTIs) can be utilized in lieu of costly and time-consuming procedures with conventional screening methods. A number of machine learning and deep learning approaches based on ligand-based and target-based approaches have been employed to predict binding affinities to save time and money in drug discovery efforts. In addition, the large chemical and genomic spaces present greater challenges as multiple drugs can be associated with multiple targets. In the perspective of medicinal chemistry, neural networks have been used in compound classification, QSAR studies, and the identification of drug targets and drug molecule’s binding modes. A variety of machine learning techniques are being used to take advantage of the large volume of complex high-dimensional information to predict interaction patterns. Here, we discuss the recent advances in DL-based DTI prediction, especially the cases relying on their 3D structural aspects.

A deep-learning approach has been applied to lead optimization in combination with traditional in silico drug discovery approaches. Wallach et al. [111] (AtomNet from Atomwise company, the first major application of DL into DTI prediction) used convolutional neural networks (CNNs) to predict the molecular bioactivity of small molecules. Based on the available complex structures of target proteins and small molecules, the binding sites are voxelized into a cube of ~20 Å. The binding site’s environment is encoded into the fixed form of the feature vectors, and then 3D CNNs are applied to the voxel volumes. Then, a binary classification model is generated to classify the input ligand as either active or inactive. More recently, Jiménez et al. [112] published another 3D-CNN-based predictor, KDEEP, using rule-based, eight pharmacophore-like descriptors obtained from the ligand–target binding site. This 3D-CNN-based scoring function achieved good performance in predicting protein–ligand absolute binding affinities on several diverse data sets. DEEPScreen [113] is a large-scale DTI prediction system using deep convolutional neural networks. The main advantage of this program is that it has readily employed available 2D structural representations of compounds at the input level. DEEPScreen learns complex features inherently from the 2D representations, producing fast and accurate predictions.

The accuracy of traditional docking modules and scoring functions can be also improved in combination with DL approaches. Morrone et al. [114] combines the docking pose rank analysis with DL, which highly improves the binding mode prediction accuracy over a baseline docking programs. Jiménez-Luna et al. [115] also successfully applied DL toward the rational molecular docking process. Similarly, deep docking [116] is developed as a deep learning platform for augmentation of structure-based drug discovery. In addition, the DL-based scoring function systems, such as ΔvinaXGB [117] of DeltaVina, CNNScore [118], and SIEVE-Score [119], are consistently reported to outperform the classical scoring methods.

## 6. Conclusions and Outlook

There have been significant advances in predicting protein CMs from the MSA of homologous proteins by analyzing the signals associated with co-evolution. Combining suitable DL methods has become a powerful framework to disentangle the underlying relationships between sequences and structural elements, leading to better drug design based on the target structures. This review covered the current trends in the protein structure prediction field, especially state-of-the-art techniques combined with deep learning architecture for contact map prediction. DL has just started to be applied to the biomolecular structure, but showed a successful strategy in the prediction field. It is fascinating and encouraging that the current DL-based techniques provide a significant advance; however, it does not mean that they ultimately “solved” the protein folding problem. Protein folding is guided and accelerated by local interactions that are rapidly formed, driving the further large-scale folding or assembly. In the folding process, some proteins need helpers like chaperones or neighboring domains. The current prediction methods do not say about this process, and we still do not have a solution to the problem of protein folding mechanism or pathway. Moreover, some proteins have floppy, ‘intrinsically disordered’ parts in their structures rather than well-defined forms. These disordered parts can act as a functional unit. DL-based approaches also showed high performance on the prediction of these regions, but they do not interpret the functional mechanism of these floppy regions. Thus, we need to develop DL approaches that will be able to address some of these caveats as well.

It is evident that the drug discovery research will continue to make progress with the learning-based approaches to explore the structures of biomacromolecules and the vast chemical space modulating these targets. In some cases, owing to the inherent limitations of data-driven research, it may be difficult to construct reliable models because of the lack of high-quality data sets. However, we believe this limitation could be overcome by incorporating expert domain knowledge and continuously growing high-quality data sets. We also expect that homology-based contact map prediction and modeling becomes more prominent to improve accuracy at a large-scale prediction problem.

## Figures and Tables

**Table 1:** List of methods for protein structure predictions. The entries consist of the most recent versions of the series.

Method/Server	Target ^a^	Topology (Incl. Earlier Steps)	Evolutionary Information (Incl. Earlier Steps)	Site
JPred	1D—SS, SA	FFNN	PSI-BLAST	http://www.compbio.dundee.ac.uk/jpred/
SSpro	1D—SS, SA(ACCpro)	BRNN–CNN	PSI-BLAST	http://scratch.proteomics.ics.uci.edu/
DISSPred	1D—SS, TA	SVM	PSI-BLAST	https://comp.chem.nottingham.ac.uk/disspred/
SPIDER3	1D—SS, SA, TA, CN	BLSTM	PSI-BLAST HHblits None (SPIDER3-single)	https://sparks-lab.org/server/spider3/
ProteinUnet	1D—SS, SA, TA, CN	CNN	None	https://codeocean.com/capsule/2521196/tree/v1
NetSurfP-2.0	1D—SS, SA, TA, DR	BLSTM	HHBlits	https://services.healthtech.dtu.dk/service.php?NetSurfP-2.0
IUPred	1D—DR	Regression	None	https://iupred2a.elte.hu/
PSIPRED	1D—SS(PSIPRED), DR(DISOPRED3) 2D—CM(MetaPSICOV2) 3D—TS(DMPfold)	FFNN	PSI-BLAST HHblits jackHMMer	http://bioinf.cs.ucl.ac.uk/psipred/
SPOT	1D—SS, SA, TA, CN(SPOT-1D), DR(SPOT-Disorder) 2D—CM(SPOT-Contact) 3D—TS(SPOT-fold)	Residual CNN BLSTM 2D-BLSTM	PSI-BLAST HHblits	https://sparks-lab.org/service/
Distill(Brewery)	1D—SS(Porter), LM(Porter+), SA(PaleAle), CN(BrownAle) 2D—CM(XX-Stout) 3D—TS(3Distill)	BRNN–CNN 2D-BRNN	PSI-BLAST HHblits	http://distillf.ucd.ie/distill/
RaptorX	1D—SS, SA, DR(RaptorX-Property) 2D—CM(RaptorX-Contact) 3D—TS(RaptorX)	CNF Residual CNN	PSI-BLAST HHblits	http://raptorx.uchicago.edu/
MULTICOM	2D—CM(DNCON2) 3D—TS	CNN	PSI-BLAST HHblits jackHMMer	http://sysbio.rnet.missouri.edu/dncon2/
TripletRes	2D—CM	Residual CNN	HHblits jackHMMer	https://zhanglab.dcmb.med.umich.edu/TripletRes/
DeepContact	2D—CM	Residual CNN	HHblits jackHMMer	https://github.com/largelymfs/deepcontact
DeepCov	2D—CM	CNN	HHblits	https://github.com/psipred/DeepCov
Pconsc4	2D—CM	CNN	HHblits	https://github.com/ElofssonLab/PconsC4
DeepCDPred	2D—MCM	FFNN	HHblits	https://github.com/PeterJamesWinn/DeepCDpred
Alphafold2	2D—MCM 3D—TS	Residual CNN	PSI-BLAST HHblits	Alphafold: https://github.com/deepmind/deepmind-research/tree/master/alphafold_casp13
Rosetta Suite	2D—MCM(trRosetta) 3D—TS	Residual CNN	PSI-BLAST HHblits	https://www.rosettacommons.org/
EVfold	3D—TS	FFNN	HHblits jackHMMer	https://v1.evcouplings.org/complex
DESTINI	3D—TS	Residual CNN	HHblits PSI-BLAST	https://sites.gatech.edu/cssb/destini/
ThreaderAI	3D—TS	Residual CNN	HHblits	https://github.com/ShenLab/ThreaderAI
NEST	3D—TS	FFNN	PSI-BLAST	http://honig.c2b2.columbia.edu/nest
C-I-TASSER	3D—TS	Residual CNN	PSI-BLAST	https://zhanglab.dcmb.med.umich.edu/C-I-TASSER/

^a^ The following abbreviations are used for targets: secondary structure (SS), solvent accessibility (SA), torsional angle (TA), contact number/density (CN), disordered region (DR), contact map (CM), multi-state contact map (MCM), and tertiary structure (TS).

## Glossary

**Sequence homology**	the biological resemblance between DNA, RNA, or protein squences, determined by their shared ancestry during the evolution of life.
**Protein co-evolution**	a statistical model that the energetic interactions between amino acids that contribute to protein structure and function can be inferred from correlations between amino acids at pairs of positions in a large selection of homologous sequences across a protein family.
**Drug target interaction (DTI)**	the binding of a drug to a target location that results in a change in its behavior/function.
**Deep learning**	a class of machine learning approach that uses artificial neural networks (ANNs) with many layers of nonlinear processing units for learning data representation.
**Contact map**	a bidimensional matrix coding the absence/presence or the probability of contact between residue pairs in a given protein.
**Direct coupling analysis (DCA)**	statistical inference framework used to infer direct co-evolutionary couplings among residue pairs in multiple sequence alignment.
**Global Distance Test (GDT) score**	ranges from 0 to 100; the percentage of amino acid residues (beads in the protein chain) within a threshold distance from the correct position; a score of ~90 GDT is informally considered to be competitive with the results obtained from experimental methods.
**Attention algorithm**	developed to mimic the way a person might assemble a jigsaw puzzle; first connecting pieces in small clumps—in this case, clusters of amino acids—and then searching for ways to join the clumps in a larger whole.
